# Mineral–Botanical Hybrid Nanofibrous Dressing for Post-Surgical Melanoma Wound Healing

**DOI:** 10.3390/polym18151857

**Published:** 2026-07-29

**Authors:** Tubanur Avci, Eray Altan, Ceren Aytar, Zehra Kanli, Elif Kaya, Serap Ayaz Seyhan, Savas Evran, Gulgun Bosgelmez Tinaz, Oguzhan Gunduz, Canan Dogan, Ahmet Akif Kızılkurtlu

**Affiliations:** 1 Department of Metallurgical and Materials Engineering, Faculty of Technology, Marmara University, Istanbul 34722, Turkey; 2 TÜBİTAK Science High School, Kocaeli 41400, Turkey; 3 Department of Biophysics, Faculty of Medicine, Istanbul Arel University, Istanbul 34010, Turkey; 4 Department of Biochemistry, Faculty of Pharmacy, Marmara University, Istanbul 34854, Turkey; 5 Department of Analytical Chemistry, Faculty of Pharmacy, Marmara University, Istanbul 34668, Turkey; 6 Department of Jewelry and Jewelry Design, Faculty of Applied Sciences, Marmara University, Istanbul 34865, Turkey; 7 Department of Basic Pharmaceutical Sciences, Faculty of Pharmacy, Marmara University, Istanbul 34668, Turkey; gulgun.tinaz@marmara.edu.tr; 8 Department of Biomedical Engineering, Faculty of Engineering and Natural Sciences, Istanbul Atlas University, Istanbul 34403, Turkey; 9 Atlas University Research Center (ARC), Atlas University, Istanbul 34408, Turkey

**Keywords:** post-surgical wound healing, *Inula helenium*, diatomite, electrospun nanofibers, dual-function biomaterial

## Abstract

Melanoma excision procedures frequently result in complex wounds that require simultaneous tumor suppression and tissue regeneration. However, current post-surgical care heavily relies on synthetic drugs, facing challenges like antibiotic resistance and delayed healing. To address this notable gap, this study introduces a novel “mineral-botanical” hybrid nanofibrous dressing to reduce reliance on conventional synthetic agents. The nanofiber surfaces were subsequently functionalized with a supercritical CO_2_ extract of *Inula helenium*, thereby introducing a highly bioactive botanical agent not previously explored in this hybrid wound-care context. Comprehensive physical and structural characterizations demonstrated that the bioactive coating serves as a resilient physical barrier, significantly enhancing the dressing’s structural integrity in fluidic environments. In vitro biological evaluations revealed a dual-action mechanism: the biomaterial exhibited excellent biocompatibility and accelerated migration in human dermal fibroblasts (HDFs), achieving nearly 100% in vitro wound closure at 24 h. Conversely, the *Inula helenium*-coated nanofibers demonstrated distinct, selective cytotoxicity against A375 melanoma cells, effectively restricting their proliferation and reducing their viability. Furthermore, the hybrid platform exhibited significant antioxidant potential and achieved 94% antibacterial inhibition against *Staphylococcus aureus*. In conclusion, this study presents a sustainable, dual-function wound dressing that suppresses residual cancer cells while promoting healthy tissue regeneration, offering a promising alternative to conventional synthetic oncological dressings.

## 1. Introduction

The global wound care market continues to expand rapidly, driven in part by the increasing incidence of cancer-related surgical interventions [1]. To provide a quantitative perspective, the global advanced wound care management market size is expected to reach $13.44 billion in 2026 and is projected to grow to $18.22 billion by 2031, reflecting the massive economic burden of wound treatments. Concurrently, the global burden of melanoma remains substantial, with over 331,000 new cases diagnosed annually worldwide, and this incidence is projected to rise to 510,000 cases by 2040 [2]. Among these, melanoma excision procedures frequently result in complex post-surgical wounds that require effective infection control and optimized healing environments. Compounding this challenge, rising antibiotic resistance has significantly reduced the effectiveness of conventional therapies for managing post-surgical wound infections, particularly in oncological settings [3]. This growing on wound-related problems has been widely characterized as a largely unrecognized global health crisis, often referred to as a “silent epidemic”, substantially impairing patients’ quality of life and prolonging recovery periods [4,5]. Inadequate post-surgical management of melanoma excision wounds can lead to delayed healing, increased infection risk, excessive scarring, prolonged hospitalization, and elevated healthcare costs, as these complex oncological wounds often fail to progress efficiently through the natural phases of tissue repair; historically, care strategies have focused primarily on wound closure rather than actively supporting the underlying biological healing processes. But growing insight into wound pathophysiology, together with advances in biopolymers and tissue engineering, has enabled the development of innovative, targeted wound dressings designed better to address the specific needs of post-surgical and melanoma-related wounds. Wound dressings are medical materials designed to protect wounds from microbial infection and to promote healing in both acute and chronic wounds. Contemporary wound dressings are broadly classified as traditional, interactive, or bioactive systems [6]. Among these, nanofiber-based dressings represent an advanced bioactive platform due to their ability to deliver antibacterial agents locally, thereby reducing microbial contamination and improving therapeutic efficacy, particularly against Gram-positive bacteria commonly implicated in chronic and open wound infections [6]. As widely reported in the literature, the inherent structural properties of nanofibrous dressings provide oxygen permeability, moisture balance, and efficient exudate management, all of which are critical factors in accelerating wound healing [7]. These nanofibrous architectures are most commonly fabricated using the electrospinning technique. This versatile and cost-effective method enables the production of continuous fibers with diameters ranging from the micro- to nanoscale [8,9,10]. Through careful adjustment of process parameters, electrospinning enables fine-tuning of fiber diameter, pore architecture, and nanofiber mat thickness, while simultaneously permitting homogeneous incorporation of bioactive agents [7,11]. This structural tunability closely mimics the native extracellular matrix (ECM), thereby enhancing cell adhesion, proliferation, and tissue regeneration. To further functionalize these electrospun matrices, manual bar coating is a critical post-processing step [10,12]. This technique utilizes a wire-wound rod to deposit a uniform layer of *Inula helenium* extract obtained via supercritical CO_2_ extraction directly onto the nanofiber surface, creating a dense bioactive film. Beyond simply loading the therapeutic agent, this coating acts as a physical barrier that modulates the nanofiber mat’s interaction with fluids; specifically, it restricts excessive water uptake and retards hydrolytic degradation. Consequently, the bar-coating process significantly enhances the dressing’s structural stability in moist wound environments, ensuring prolonged mechanical integrity.

Nanofibers can be fabricated from a wide range of natural and synthetic polymers. Among these natural polymers, Polyvinyl alcohol (PVA) has emerged as a particularly advantageous synthetic polymer for electrospun wound dressing applications due to its excellent water solubility, film-forming ability, and electrospinnability [13]. Unlike many hydrophobic synthetic polymers that require organic solvents, PVA can be processed in aqueous systems, reducing toxicity concerns and enhancing environmental sustainability [14]. Furthermore, PVA exhibits high mechanical flexibility, biocompatibility, and chemical stability, while its abundant hydroxyl groups facilitate hydrogen bonding and the efficient loading of bioactive compounds. Essentially, this PVA chemistry also enables effective tailoring with other bio-sourced materials—such as crystalline cellulose or cellulose nanocrystals—significantly enhancing interfacial interactions at the nanoscale [15] and thereby improving the overall thermal, mechanical, and structural properties of the resulting composites [16]. This strong interfacial bonding capacity directly strengthens the rationale for our pursued methodology, which efficiently integrates the PVA matrix with natural calcined diatomite and botanical agents. Calcium ions (Ca^2+^) have been reported to play a critical role in platelet activation and the blood coagulation cascade, thereby accelerating the early hemostatic phase of wound healing [17]. In addition, aluminosilicate-based materials containing aluminum oxide (Al_2_O_3_) have been shown to enhance surface-mediated clot formation by promoting protein adsorption and platelet adhesion at the wound interface [18]. Diatomite, a naturally occurring porous silica-based mineral, has gained increasing attention as a biocompatible and sustainable alternative to synthetic silica nanoparticles due to its high surface area, adsorption capacity, and mineral-rich composition [19,20]. Calcination improves the purity and structural stability of diatomite while increasing Ca^2+^ availability, which, based on existing literature, has the potential to support rapid hemostasis at the wound site [19,21]. These characteristics suggest that locally sourced diatomite offers potential hemostatic and regenerative properties while reducing reliance on costly synthetic nanomaterials associated with environmental and biocompatibility concerns, representing a sustainable strategy for advanced wound-dressing applications [4,5].

Plants have been widely used in traditional medicine due to their rich content of bioactive secondary metabolites, particularly flavonoids and sesquiterpene lactones, which exhibit diverse biological activities [22]. *Inula helenium* L. (Asteraceae) has long been used in traditional medicine for respiratory, gastrointestinal, and dermatological disorders [23]. Contemporary studies confirm that *Inula helenium* is rich in flavonoids and eudesmane-type sesquiterpene lactones, with alantolactone and isoalantolactone identified as its high bioactive components [24]. These compounds have demonstrated antiproliferative and apoptosis-inducing effects in various cancer cell lines and have been shown to suppress tumor growth and enhance antitumor immune responses [25]. *Inula helenium* bioactive compounds were obtained via supercritical CO_2_ extraction, a solvent-free, low-temperature technique that preserves thermolabile constituents and enhances extract purity and bioactivity compared with conventional extraction methods. By minimizing thermal degradation and residual solvent contamination, supercritical extraction improves the safety and efficacy of the resulting extracts, making *Inula helenium* a promising natural source for advanced wound dressing systems designed to support post-surgical melanoma wound healing through combined antioxidant, anti-inflammatory, and antiproliferative effects.

In this study, calcined diatomite was incorporated into a PVA-based electrospun fibrous matrix to create a multifunctional nanofiber mat, in which the intrinsic Al_2_O_3_ and porous silica structure of diatomite are expected to enhance hemostasis and serve as a natural nanocarrier. This mineral-based matrix was subsequently surface-coated with supercritical CO_2_ extracted from *Inula helenium*, creating a dual-function system that is theoretically designed to leverage the well-documented hemostatic potential of diatomite while enabling sustained bioactive release. Furthermore, the mechanical integrity of the designed mats was rigorously evaluated through comparative experimental tensile tests and numerical simulations to predict stress distribution under physiological loads. While the current literature abounds with nanofibrous dressings, there remains a notable gap in natural-based platforms that utilize mineral and botanical agents to provide potential hemostatic and bio-regenerative effects, capable of simultaneously managing the complex microenvironment of oncological wounds and minimizing reliance on synthetic drugs. Addressing this limitation, the present work introduces a novel ‘mineral-botanical’ hybrid platform that offers a bio-based alternative to synthetic drugs. Therefore, this research addresses the need for multifunctional dressings in oncological surgery by synergizing the theoretical hemostatic properties of calcined diatomite with the beneficial bioactivity of *Inula helenium*, suggesting that locally sourced natural hybrids can serve as promising, cost-effective alternatives to conventional synthetic dressings. While plant-extract-loaded electrospun mats have been widely reported, predominantly focusing on broad-spectrum antibacterial purposes, the novelty of our specific biomaterial design is threefold: (I) it utilizes a surface bar-coating strategy that acts as a physical barrier against rapid hydrolytic degradation, maintaining structural integrity in moist environments without requiring toxic chemical crosslinkers; (II) it incorporates calcined diatomite, a highly porous and sustainable biosilica, to establish a bio-based ‘mineral–botanical’ hybrid matrix that eliminates the reliance on potentially cytotoxic synthetic metallic or inorganic nanoparticles; and (III) it addresses the specific challenges of post-surgical oncological wounds by exhibiting a dual-action ‘selective cytotoxicity’—actively suppressing A375 melanoma cells while simultaneously accelerating healthy human dermal fibroblast (HDF) migration [26,27,28,29].

## 2. Materials and Methods

### 2.1. Materials

PVA (Mw = 89,000–98,000, 99% hydrolyzed) was purchased from Sigma Aldrich (Gillingham, UK). *Inula helenium* (1 kg) was purchased from a local herbal store in Manisa (Turkey). Isopropyl alcohol (IPA, purity 99%) was purchased from Sisco Research Laboratories (Mumbai, India). Pure diatomaceous earth was purchased from a commercial supplier (Pars, Turkey). Dimethyl sulfoxide (DMSO) was obtained from Sigma–Aldrich (St. Louis, MO, USA). Tween 80 was purchased from Merck KGaA, Darmstadt, Germany.

### 2.2. Supercritical Fluid Extraction of Inula helenium

*Inula helenium* leaves were ground into a fine powder using a mortar and pestle to enhance the extraction efficiency. The extraction process was carried out using a supercritical fluid extraction system (SUPEREX F-500 model, Superex, Konya, Turkey) utilizing carbon dioxide (SC-CO_2_) as the solvent. A total of 100 g of powdered plant material was loaded into the extraction vessel. The extraction parameters were maintained at 275 bar and 50 °C for 3 h. The resulting extract was collected and stored at +4 °C to prevent degradation. The extraction process yielded 1.8 g of crude extract from 100 g of plant powder, corresponding to an extraction yield of 1.8% (*w*/*w*).

### 2.3. Electrospinning Process

Before fabrication, raw diatomite was calcined to remove organic impurities, reduce moisture content, and enhance its thermal stability and compatibility with the polymer matrix. Diatomite was placed in a ceramic crucible and calcined in a muffle furnace at 600 °C for 1 h. The calcined diatomite was allowed to cool at room temperature for 30 min, then passed through a 100-mesh sieve to remove agglomerates.

To prepare the base polymer solution for electrospinning, PVA was dissolved in distilled water (13% *w*/*v*) under continuous magnetic stirring at 120 °C for 1 h to ensure complete dissolution and homogeneity. Upon cooling the solution to room temperature, DMSO was added as a cosolvent at 1% *v*/*v*. Subsequently, calcined diatomite (5% *w*/*w* relative to the polymer) and Tween 80 (3% *v*/*v*) were incorporated into the mixture. Tween 80, a non-ionic surfactant, was used to reduce surface tension and facilitate the formation of a stable Taylor cone during electrospinning [8]. The final composite suspension was stirred magnetically for an additional 30 min to ensure uniform dispersion before electrospinning.

Electrospinning was performed using the prepared composite gel solution. The process was carried out with a 17-gauge needle at 25.2 kV and a flow rate of 1 mL/h. Electrospinning was conducted at ambient conditions (25 ± 2 °C, relative humidity 40–50%) using a tip-to-grounded collector (covered with wax paper) at a distance of 15 cm. Electrospinning was maintained for 3 h, resulting in the successful formation of nanofibers. Upon completion, the device was turned off, and the wax-paper-covered collector was carefully removed. The thin fiber layer was collected, with a portion reserved for further analysis and the remainder stored at room temperature for subsequent bar-coating applications.

### 2.4. Manual Bar Coating Application

The obtained extract was dissolved in 45 mL of IPA at room temperature to a final concentration of 40 mg/mL (4% *w*/*v*) for coating nanofibers. The prepared *Inula helenium* solution was applied to the PVA/diatomite nanofiber membrane surfaces using a manual bar coating device (No. 4 bar, stainless-steel spiral wire, 40 µm wet film thickness; RK Print Coat Instruments Ltd., Royston, UK). Both sides of the nanofibers were coated twice, with approximately 1 mL of solution applied per layer in each coating step. To minimize variations in coating thickness, the coating rod was manually drawn at a constant speed and uniform pressure. Based on the applied volume (~1 mL per layer) and the extract concentration (40 mg/mL), the theoretical dry coating mass was calculated as approximately 40 mg per layer, resulting in a total theoretical final dry coating mass of ~160 mg for the fully coated sample (two layers on both sides). After each layer, the samples were dried in an oven at 40 °C for 10 min to evaporate the IPA. Coating uniformity was initially verified by visual inspection to ensure a surface free of cracks or air bubbles, and subsequently confirmed at the microstructural level via SEM imaging (as discussed in Section 3.1). The fabrication of PVA/diatomite nanofibers followed by their coating with *Inula helenium* supercritical CO_2_ extract is illustrated in Figure 1.

### 2.5. Scanning Electron Microscopy (SEM)

The surface morphology and microstructural features of the fabricated nanofibers were examined using scanning electron microscopy (SEM) (ZEISS, Jena, Germany) at an accelerating voltage of 10.00 kV. Before imaging, all nanofiber samples were sputter-coated with a thin layer of gold–palladium (Au/Pd) for 120 s using a sputter coater (Quorum Technologies, Lewes, UK.) to minimize electrostatic charging and enhance image resolution. Fiber diameter measurements were performed by analyzing SEM micrographs with ImageJ (v1.47; National Institutes of Health, Bethesda, MD, USA). For each sample, at least 100 individual fibers were measured, and the obtained data were used to generate fiber diameter distribution histograms.

### 2.6. Fourier Transform Infrared Spectroscopy (FTIR)

Fourier transform infrared spectroscopy (FTIR) analysis was performed using an FT/IR-4700 spectrometer (JASCO Corporation, Tokyo, Japan) to qualitatively investigate the chemical structure and functional groups of the nanofiber samples. The FTIR spectra were recorded in the wavenumber range of 4000–400 cm^−1^ at room temperature, with a spectral resolution of 4 cm^−1^. For each sample, 32 scans were accumulated to improve the signal-to-noise ratio.

### 2.7. Differential Scanning Calorimetry (DSC)

To determine thermal properties, a DSC-60 Plus calorimeter (Shimadzu, Japan) was used to monitor changes in the heat capacity of the nanofiber mats as temperature increased. Each nanofiber mat was weighed to approximately 5 mg and sealed in a standard aluminum pan before analysis. All measurements were carried out over the temperature range of 25 °C to 300 °C at a constant heating rate of 20 °C/min, without an inert nitrogen atmosphere, to directly assess the thermal response of the samples under these conditions. Then, corresponding thermograms were recorded for further evaluation.

### 2.8. Mechanical Analysis of the Nanofiber Mats

The mechanical properties of the nanofiber mats were evaluated using a universal testing machine (Shimadzu EZ-X, Tokyo, Japan) with a maximum load capacity of 5 kN. Rectangular samples measuring 10 × 50 mm were prepared from each nanofiber mat and tested at a crosshead speed of 5 mm/min. Three replicate samples were tested at room temperature, and the obtained data were used to calculate the mechanical properties of the nanofiber mats [30]. The mechanical properties, such as tensile stress, strain, and modulus of elasticity, can be determined utilizing Equations (1), (2), and (3), respectively [31]

(1) $$\sigma_{T}=\frac{F}{A}$$

(2) $$\epsilon =\frac{\delta }{L}$$

(3) $$E=\frac{\sigma_{T}}{\epsilon }$$

where F, A, δ, L, and ϵ express the axial force, cross-sectional area, deformation, length, and strain, respectively.

#### Numerical Analysis of the Nanofiber Mats

Numerical analyses were conducted to assess the stress behavior and total deformation of the *Inula helenium*-coated PVA/diatomite and PVA/diatomite mats based on finite element method and each mat was modelled under fixed boundary conditions. Numerical modelling of all mats was determined utilizing ANSYS Workbench 2020 R2 (Canonsburg, PA, USA). The von Mises and normal stress analyses were numerically performed to validate the experimental tensile stress. The experimental dimensions of the mats were utilized in the numerical modelling of the tensile stress. In addition, the pressure stress and total deformation behaviors were carried out to determine the effect of the pressure and material type on the mats and the dimensions for width, length, and thickness of the mats were considered to be 50 mm, 50 mm and 0.03 mm, respectively. The forces for tensile tests were applied along the *Y*-axis, whereas the pressures for equivalent stress and total deformation were implemented to *Z*-axis. The modulus of elasticity for *Inula helenium*-coated PVA/diatomite and PVA/diatomite mats was experimentally calculated as 64.62 MPa and 75.33 MPa, respectively. Ux, Uy, and Uz for fixed end were used to be 0 mm and the element size for meshing were determined as 0.1 mm.

### 2.9. Swelling-Degradation Test

Swelling and degradation tests were performed to evaluate the water uptake behavior and mass loss of the fabricated nanofibers. Phosphate-buffered saline (PBS, pH 7.4) was employed as the incubation medium to simulate physiological conditions. For the swelling study, the initial dry weight of each sample (W*_d_*) was recorded. The samples were then immersed in 1 mL of PBS in Eppendorf tubes and incubated in a thermal shaker at 37 °C. At predetermined intervals (every 24 h) for up to 10 days, the samples were removed, and excess surface liquid was gently blotted with filter paper. The swollen weight (W*_w_*) was measured, and the swelling ratio was calculated using Equation (4) [32]

(4) $$S=\frac{W_{w}-W_{d}}{W_{d}}*100$$

For the degradation study, samples with a known initial dry weight (W_0_) were immersed in 1 mL of PBS and incubated under identical conditions. Every 24 h for 5 days, the samples were removed from the PBS solution and dried at 37 °C for 24 h. The final dried mass (W*_t_*) was then recorded. The degradation rate (weight loss %) was determined according to Equation (5)

(5) $$D=\frac{W_{0}-W_{t}}{W_{0}}*100$$

### 2.10. Determination of Total Phenolic Content (TPC Analysis)

Total phenolic content (TPC) of nanofibers was determined spectrophotometrically according to a modified method using Folin–Ciocalteu reagent. Firstly, nanofiber samples (0.01 g) were extracted with %70 ethanol (*v*/*v*) using an orbital shaker incubator (BIOSAN ES-20 Riga, Latvia) at a constant stirring speed (200 rpm, at 37 °C) for 1 h. Briefly, 100 μL of the sample was added to 4.0 mL of distilled water and 100 μL of Folin–Ciocalteu reagent, and the mixture was then kept at 30 °C for 5 min. 800 μL of 6% Na_2_CO_3_ solution was added, and the mixture was incubated at 30 °C for 30 min. The blue coloration produced by the reduction in the Folin–Ciocalteu reagent by phenolic compounds was measured at 688 nm using a UV–Vis spectrophotometer (UV-1601, Shimadzu Co., Ltd., Kyoto, Japan), yielding a significant absorbance indicative of antioxidant potential. Results are expressed as gallic acid equivalents (GAE, mg GA per 1 mL sample). The calibration equation for GA is A = 0.0015x + 0.0273 (R^2^ = 0.9959). Each test was repeated three times [33].

### 2.11. In Vitro Evaluation of HDF and A375 Cell Behavior on PVA/Diatomite-Based Nanofibers

Human dermal fibroblast (HDF) cells (PCS-201-012™, ATCC, Manassas, VA, USA) and human melanoma (A375) cells (CRL-1619™, ATCC, USA) were used to evaluate the biocompatibility, adhesion, and migration behavior on PVA/diatomite-based nanofibers. In this study, the term “PVA/diatomite-based nanofibers” refers to both uncoated and *Inula helenium*-coated PVA/diatomite nanofibers. The cells were cultured in Dulbecco’s Modified Eagle Medium (DMEM, Gibco, Grand Island, NY, USA) supplemented with 10% fetal bovine serum (FBS, Gibco, USA) and 1% penicillin–streptomycin (Gibco, USA). All cultures were maintained at 37 °C in a humidified atmosphere containing 5% CO_2_. Before cell seeding, all nanofiber samples were sterilized under ultraviolet (UV) irradiation for 15 min.

### 2.12. MTT Cell Viability Assay

Cell viability on PVA/diatomite-based nanofibers was quantitatively assessed using the MTT Cell Proliferation and Cytotoxicity Assay Kit (Elabscience, Houston, TX, USA). This assay is based on the reduction of MTT by mitochondrial dehydrogenases into purple formazan crystals, which reflects the metabolic activity of viable cells. HDF and A375 cells were seeded onto the sterilized nanofiber scaffolds in 96-well plates at densities of 5 × 10^3^ cells per well and incubated for 1, 3, and 7 days. At each time point, the culture medium was replaced with 100 µL of fresh DMEM containing 50 µL of MTT reagent, then incubated at 37 °C for 2.5 h. The resulting formazan crystals were dissolved in dimethyl sulfoxide (DMSO; Sigma-Aldrich, St. Louis, MO USA) and incubated at 37 °C for an additional 10 min, as previously described [34,35]. Absorbance was measured at 570 nm using a microplate reader (Thermo Multiskan SkyHigh, Waltham, MA, USA).

### 2.13. Scratch (Wound Healing) Assay

A scratch (wound healing) assay was conducted to assess the effects of PVA/diatomite-based nanofibers on the migratory behavior of HDF and A375 cells. Briefly, HDF and A375 cells were seeded separately in 24-well plates at a density of 2.5 × 10^4^ cells per well and cultured until approximately 90% confluence. A linear scratch was generated in each cell monolayer using a sterile 200 µL pipette tip. After washing the wells gently with phosphate-buffered saline (PBS, Gibco, USA) to remove detached cells, fresh DMEM was added. Sterilized nanofiber discs corresponding to the experimental groups were then placed into the wells. Wound images were acquired at 0 and 24 h for HDF cells and at 0, 24, and 48 h for A375 cells using an inverted phase-contrast microscope (Olympus IX73, Tokyo, Japan). Wound closure was quantitatively analyzed using ImageJ software [36].

### 2.14. Live/Dead Assay

The viability and cytotoxicity of HDF and A375 cells cultured on PVA/diatomite-based nanofibers were evaluated using the LIVE/DEAD Viability/Cytotoxicity Kit (Invitrogen, Thermo Fisher Scientific, USA), according to the manufacturer’s instructions. The Live/Dead staining assay was performed on day 3 of the cell culture period. Briefly, at the end of day 3, the samples were washed three times with phosphate-buffered saline (PBS) to remove residual medium and then incubated with the staining solution for 30 min at room temperature in the dark. After staining, the samples were examined under a fluorescence microscope (Invitrogen EVOS M5000, Waltham, MA, USA) at 20× magnification. Viable cells were identified by green fluorescence, whereas non-viable cells were identified by red fluorescence.

### 2.15. Statistical Analysis

All in vitro experiments were performed with at least three independent biological replicates (*n* = 3). Quantitative data obtained from MTT absorbance measurements, wound closure analysis, and Live/Dead Assay were analyzed using GraphPad Prism software (version 8.0.2, GraphPad Software, Boston, MA, USA) or equivalent statistical software. Depending on the experimental design, one-way or two-way analysis of variance (ANOVA) was used for group comparisons, followed by appropriate post hoc multiple-comparison tests (Tukey’s and/or Dunnett’s) when statistical significance was observed. Data were expressed as mean ± standard error of the mean (SEM), and statistical significance was accepted at *p* < 0.05.

### 2.16. Antibacterial Activity Test

Antibacterial activities of *Inula helenium* extracts against *Staphylococcus aureus* (*S. aureus*) ATCC 25923, *Escherichia coli* (*E. coli*) ATCC 25922, and *Pseudomonas aeruginosa* (*P. aeruginosa*) were tested using the well diffusion test performed on ATCC 27853. Overnight bacterial cultures were spread onto Mueller-Hinton Agar plates after being adjusted to McFarland 0.5 density. *Inula helenium* extracts with concentrations of 17 mg/mL, 34 mg/mL, and 68 mg/mL were added to wells cut to a depth of 6 mm. Then, it was incubated at 37 °C overnight. Inhibition zones were determined by measuring the diameters of the zones formed after incubation. A gentamicin (GEN) 10 µg disc was used as the positive control, according to the CLSI-specified dose.

The antibacterial activities of the PVA/diatomite nanofiber and *Inula helenium*-coated PVA/diatomite nanofiber samples against *S. aureus*, *E. coli*, and *P. aeruginosa* were tested using the liquid microdilution method, modified as described by the Clinical Laboratory Standards Institute (CLSI) [37]. In a 48-well plate, fiber samples were added to LB liquid medium. The bacterial culture was added to the medium to achieve a final concentration of 5 × 10^5^ CFU/mL in the wells. It was incubated overnight at 37 °C in a shaking incubator. Bacterial growth inhibition was then determined by measuring bacterial density at 625 nm with a spectrophotometer. The experiment was performed in triplicate. The inhibition rate was compared with the control group (without added fiber) and calculated using the following formula:

% Inhibition: (ODcontrol-ODsample)/ODcontrol × 100.

ODcontrol: Control group optical density (no fiber added group).
ODsample: Sample group optical density.

## 3. Results

### 3.1. SEM Investigation

The surface morphology of electrospun membranes was investigated using SEM to examine the structural properties of PVA/diatomite nanofibers and to evaluate the effect of an *Inula helenium* extract coating on these structures. As shown in Figure 2, the PVA/diatomite nanofibers (Figure 2b) exhibited a continuous and randomly oriented fibrous network with a uniform and bead-free fiber formation. Based on the diameter distribution histogram (Figure 2c), the average fiber diameter was calculated to be approximately 544 nm, indicating a relatively homogeneous size distribution. Consistent with Akduman’s (2019) findings, which reported that diatomite loading preserved fiber continuity and produced bead-free structures, our results confirm that integrating diatomite into the polymer matrix maintained electrospinning stability [38]. In contrast, the *Inula helenium* -coated PVA/diatomite nanofibers (Figure 2a) displayed a noticeably denser morphology with surface coverage and partial pore filling. This structural change confirms the successful deposition of the bioactive layer from *Inula helenium* onto the nanofiber surface. While diatomite loading alone has been reported to reduce mean fiber diameters and significantly affect surface texture, the observed pore masking and surface densification in this study are primarily attributed to the post-fabrication bar-coating process rather than to electrospinning instabilities.

### 3.2. FTIR Spectroscopy Analysis

FTIR spectroscopy presented the chemical composition of the PVA/diatomite and *Inula helenium*-coated PVA/diatomite nanofiber structures, as demonstrated by the spectra presented in Figure 3.

In the pure PVA spectrum, the broad band in the 3500–3200 cm^−1^ range represents O–H stretching, the peak at 2920 cm^−1^ represents aliphatic C–H stretching, and the peak at 1730 cm^−1^ represents C=O stretching of residual acetate groups resulting from incomplete hydrolysis [39]. The calcined diatomite spectrum is dominated by strong bands in the 1100–1000 cm^−1^ region, which correspond to Si–O–Si asymmetric stretching vibrations, a fundamental characteristic of the siliceous structure [38]. In the spectrum of the *Inula helenium* extract, peaks of 3500–3200 cm^−1^ (O–H) and 2920 cm^−1^ (C–H stretching of CH_2_ groups) were observed. Specifically, the peak around 800 cm^−1^ in the fingerprint region confirms the presence of β-type glycosidic bonds and a 2-ketofuranose sugar structure in the composition, consistent with the literature [40]. In the PVA/diatomite nanofiber spectrum, the simultaneous presence of both C–H (~2920 cm^−1^) bands from PVA and Si–O–Si (~1100 cm^−1^) bands from diatomite demonstrates the successful integration of the inorganic phase into the polymer matrix. In the *Inula helenium*-coated PVA/diatomite nanofiber, the broadening of the band in the 3200–3500 cm^−1^ range indicates the addition of phenolic and alcoholic –OH groups from the extract to the surface. Furthermore, the new peak appearing around ~1650 cm^−1^ is attributed to C=O and aromatic C=C vibrations in the extract’s structure. The absence of a characteristic peak indicating the formation of a new covalent bond suggests that the extract adheres to the nanofiber surface via physical adsorption and hydrogen bonds rather than chemical bonding. These findings are consistent with literature data on electrospinning post-coating systems containing plant extracts.

### 3.3. Differential Scanning Calorimetry

The DSC thermogram shown in Figure 4 compares the thermal behavior of the PVA/diatomite nanofiber mat (red curve) with that of the *Inula helenium*-coated PVA/diatomite nanofiber mat (blue curve) over the temperature range of 25–300 °C.

The gradually decreasing heat-flow signal observed for the PVA/diatomite nanofiber indicates a continuous endothermic process associated with softening, melting of crystalline regions, and the onset of thermal degradation of the PVA matrix under non-inert conditions, consistent with previously reported PVA systems [13]. Both samples display a clear endothermic phase in the 215–230 °C region, corresponding to a major transition in which melting and thermo-oxidative decomposition of PVA chains occur; similar transition temperatures have been reported for electrospun PVA nanofibers and PVA composites [41]. In the coated nanofiber mat, this event occurs at 215.87 °C, slightly lower than the 228.41 °C peak observed for the PVA/diatomite nanofiber, suggesting that the coating affects chain mobility and crystalline packing, thereby triggering the main transition earlier [42,43]. At higher temperatures, the *Inula helenium*-coated PVA/diatomite nanofiber shows a less pronounced endothermic slope than the PVA/diatomite sample, indicating a modified thermal behavior introduced by the coating layer. Taken together, these findings indicate that the surface coating shifts the principal thermal transition to a slightly lower temperature and influences the general thermo-oxidative response of the nanofiber mats at elevated temperatures.

### 3.4. Mechanical Analysis of the Nanofiber Mats

Tensile testing revealed distinct mechanical behaviors between the *Inula helenium*-coated PVA/diatomite nanofiber mats and PVA/diatomite nanofiber mats. As summarized in Table 1, tensile testing revealed distinct mechanical behaviors between the groups. The *Inula helenium*-coated PVA/diatomite mat exhibited lower maximum force, stress, stroke, and strain values compared to the uncoated mats, indicating a stiffer and less extensible structure. In contrast, the PVA/diatomite mat exhibited higher mechanical performance, indicating more ductile and deformable behavior. The underlying material architecture can explain this difference: compared to the PVA/diatomite mats, the *Inula helenium*-coated group exhibited a more brittle mechanical response, which can be attributed to the formation of a stiff, low-ductility organic coating layer on the mat surface [44]. This inula-based layer likely behaves in a glassy manner, restricting polymer chain mobility at the interface and promoting early crack initiation and propagation, resulting in low elongation at break and sudden failure [45]. Consistently, the decreased modulus of elasticity observed in the coated system further confirms this distinct mechanical behavior.

In contrast, the PVA/diatomite system provides a more ductile matrix; the hydrophilic and partially flexible PVA chains can undergo segmental motion, chain stretching, and intermolecular slippage before fracture, allowing higher strain and energy absorption [13,46], while the porous, high–surface-area diatomite particles dispersed within the PVA matrix contribute to improved load transfer and crack deflection, acting as micro-stress redistributors and promoting mechanisms such as particle pull-out and micro-yielding rather than catastrophic crack growth [47,48]. Together, these features result in a composite structure that can accommodate larger deformations under tensile loading, explaining the markedly higher stroke and strain values observed for the PVA/diatomite mats compared to the more rigid *Inula helenium*-coated counterparts. The mechanical testing revealed clear differences between the *Inula helenium*-coated PVA/diatomite nanofiber mats and the PVA/diatomite mats. The coated samples exhibited lower maximum force (0.79 ± 0.03 N) and maximum stress (2.94 ± 0.57 MPa) compared to the PVA/diatomite mats (1.10 ± 0.16 N and 3.90 ± 0.31 MPa, respectively). In addition, the coated mats showed reduced extensibility, as indicated by their lower maximum stroke (2.04 ± 0.64 mm vs. 6.75 ± 3.56 mm) and maximum strain (4.05 ± 1.26% vs. 13.56 ± 7.33%). Consistently, the elastic modulus was higher for the PVA/diatomite group (75.33 MPa) than for the *Inula helenium*-coated mats (64.62 MPa), confirming that the incorporation of PVA/diatomite without the herbal coating yields mechanically stronger and more extensible nanofiber constructs suitable for tissue engineering and topical biomedical applications [49,50,51,52].

#### Numerical Analysis of the Nanofiber Mats

According to Table 2, based on experimental and numerical methods, the highest stress was obtained using the PVA/diatomite nanofiber mat. In contrast, the lowest stress was determined for the *Inula helenium*-coated PVA/diatomite nanofiber mat. This finding may be directly related to the tensile force and the mats’ cross-sectional area, and an increase in the mats’ mechanical properties may affect the tensile force. For mats with equal cross-sectional area, a high tensile force can lead to a high stress on the mats. In the experimental tensile tests, the tensile force applied to the *Inula helenium*-coated PVA/diatomite nanofiber mat was lower than that applied to the PVA/diatomite nanofiber mat, supporting the observed difference in stresses. The maximum differences between experimental and numerical stresses were found to be ±1.48% for the *Inula helenium*-coated PVA/diatomite nanofiber mat and ±0.27% for the PVA/diatomite nanofiber mat. This low percentage difference may indicate that the mat production and experimental tensile testing were carried out with high accuracy. The von Mises stress and normal stress in the numerical analyses were found to be equal, and the analysis contours are illustrated in Figure 5.

Figure 5a shows the mesh quality according to the skewness approach, with data ranging from 1.3 × 10^−10^ to 1.4 × 10^−10^. Figure 5b and 5c illustrate the von Mises stresses of the *Inula helenium*-coated PVA/diatomite nanofiber mat and the PVA/diatomite nanofiber mat, respectively. In contrast, Figure 5d,e demonstrates their normal stresses along the *Y*-axis. Stress distributions on the mats were observed to be homogeneous for both stress types, while low levels were detected in regions near the fixed ends. Numerical analyses were performed to examine the effects of pressure and mat type on equivalent stress and total deformation. Numerical equivalent stresses and total deformations were listed in Table 3.

As shown in Table 3, increasing pressure increases equivalent stress, while mat type has no effect. This finding suggests that stress behavior may not be related to the mats’ modulus of elasticity. In addition, an increase in pressure increases the total deformation. Under the same pressures, the *Inula helenium*-coated PVA/diatomite nanofiber mats exhibit more deformation compared to the PVA/diatomite nanofiber mats. The mats’ modulus of elasticity can explain this finding. An increase in the modulus of elasticity leads to a decrease in deformation [53,54]. Numerical contours for equivalent stress and total deformation were presented in Figure 6 and Figure 7, respectively.

Figure 6a–h show that the minimum stress was observed in the middle areas of the mats, while the maximum stress was detected at the edges. Negative stresses were also monitored at the corners. This finding may suggest that regions exposed to the highest stress are also the most affected. Figure 7a–h shows that the highest total deformation was observed in the middle regions of the mats, whereas the lowest was observed at the edges. This finding may support that the central region of the mat is the area subjected to the most pressure.

Analysis of variance (ANOVA) was used to determine the effects of pressure and mat type on the numerical equivalent stress and total deformation. The analysis was conducted at the 95% confidence level using Minitab R15, and the results are presented in Table 4.

According to the ANOVA results in Table 4, pressure has an effect on the equivalent stress, while the mat type has no effect. This finding is consistent with the numerical analyses shown in Table 3. Furthermore, the most effective parameters for the total deformation of the mats were pressure (96.61% effect) and mat type (2.83% effect).

### 3.5. Swelling—Degradation Test Results

Figure 8 presents a comparative analysis of the time-dependent swelling and degradation behavior of PVA/diatomite and *Inula helenium*-coated PVA/diatomite nanofibers. PVA/diatomite nanofibers exhibited a high swelling ratio of approximately 95% on the first day of incubation. This high level was maintained relatively well between days 1 and 5. Still, a significant decrease in the swelling rate was observed from day 6 onwards, attributable to the gradual loss of structural integrity. The high hydrophilicity, caused by the hydroxyl groups in the PVA structure and the porous structure of diatomite, plays a critical role in promoting water uptake and retention within the nanofiber matrix [55,56]. In contrast, *Inula helenium*-coated PVA/diatomite nanofibers showed significantly lower swelling rates throughout the entire incubation period. This rate, which was at 30–35% on the first day, steadily decreased, falling below 5% on days 7–10. While the initial 30–35% swelling is sufficient to manage the heavy exudate typical of the early post-surgical inflammatory phase, the subsequent reduction in water uptake is a crucial protective mechanism to prevent rapid hydrolytic dissolution of the matrix during long-term application. This demonstrates that the extract coating acts as an effective physical barrier by restricting direct contact between the hydrophilic groups on the PVA surface and water [57]. This physical barrier effect of the botanical coating eliminates the need for conventional, potentially cytotoxic chemical crosslinking agents, thereby maintaining the dressing’s high biocompatibility. The time-dependent degradation profile of the nanofibers correlates directly with their swelling behavior. PVA/diatomite samples reached a mass loss of 75% on day 3 due to rapid water ingress into the matrix and hydrolytic dissolution of polymer chains. On the other hand, *Inula helenium*-coated PVA/diatomite samples showed a more stable degradation profile, with mass losses of approximately 58% on day 3 and 65% on day 5. Silica-based additives such as diatomite are reported in the literature to increase water absorption, making the matrix more vulnerable to hydrolytic degradation [55,58]. The coating method applied in this study was found to slow down the degradation kinetics by preventing water penetration. The obtained low swelling index and controlled degradation profile strengthen the medical application potential of the developed nanofibers. Particularly in postoperative wound settings and on mobile body joints, this structure, which can maintain its shape for extended periods without immediate loss, emerges as a promising candidate for durable wound dressing. Although the organic coating introduces brittleness under dry testing conditions, the physiological moist wound environment compensates for this limitation by preventing rapid hydrolytic degradation, thereby allowing the dressing to conform physically and sustain the mechanical integrity critical for tissue healing.

### 3.6. Determination of Total Phenolic Content (TPC Analysis)

Oxidative stress is widely recognized as a critical factor that disrupts the normal wound healing cascade by prolonging inflammation, inducing cellular damage, and delaying tissue regeneration. Excessive accumulation of reactive oxygen species (ROS) in chronic or surgically induced wounds can impair fibroblast proliferation, inhibit keratinocyte migration, and disrupt extracellular matrix remodeling, ultimately slowing epithelialization. Therefore, advanced wound-dressing materials are expected not only to act as physical barriers but also to actively regulate oxidative stress in the wound microenvironment. This aspect becomes particularly important in the context of surgical wounds following melanoma excision, where large tissue defects are often created during tumor removal. Post-surgical melanoma wounds are frequently associated with elevated oxidative stress and inflammatory responses, which may delay tissue regeneration and compromise the healing process. Consequently, biomaterials capable of modulating ROS levels may provide significant therapeutic benefits in supporting post-operative wound healing.

The total phenolic content was determined quantitatively using the Folin–Ciocalteu reagent, with Gallic acid as the standard. The total phenolic content was calculated with the help of the graph shown in Figure 9a, and the standard curve equation was y = 0.0015x + 0.0273, where R^2^ = 0.9959. As shown in Figure 9b, PVA/diatomite nanofibers exhibited negligible antioxidant potential, which can be attributed to the chemically inert nature of PVA and the limited presence of redox-active functional groups in diatomite. In contrast, a pronounced increase in total phenolic content was observed for the *Inula helenium*-coated PVA/diatomite nanofiber, acting as a strong indicator of its antioxidant potential and confirming that the plant-derived coating successfully introduced bioactive phytochemicals into the nanofiber structure. Previous studies have reported that *Inula helenium* contains a variety of phenolic and flavonoid compounds, including chlorogenic acid, caffeic acid, rutin, quercetin, luteolin, and kaempferol, which are known for their strong antioxidant and free-radical-scavenging activities [59,60,61,62]. These phenolic constituents can neutralize reactive oxygen species through electron or hydrogen donation mechanisms, thereby reducing oxidative damage at the cellular level. Furthermore, plant-derived phenolic compounds have been widely reported to promote wound healing by reducing intracellular ROS levels, stimulating fibroblast proliferation, and enhancing collagen synthesis during the proliferative phase of wound repair. In the context of melanoma surgery, such antioxidant potential may be particularly beneficial for maintaining cellular homeostasis in the wound microenvironment and supporting tissue regeneration following tumor excision. Overall, the significant increase in antioxidant potential observed in the *Inula helenium*-coated PVA/diatomite nanofiber demonstrates that plant-based functionalization effectively enhances the bioactivity of the nanofiber scaffold. Therefore, this multifunctional material may serve as a promising post-surgical wound dressing for melanoma excision sites, capable of mitigating oxidative stress and promoting tissue regeneration during healing.

### 3.7. In Vitro Biocompatibility and Cellular Response of PVA/Diatomite-Based Nanofibers

The in vitro biocompatibility and selective cytotoxicity of the PVA/diatomite and *Inula helenium*-coated nanofibers were evaluated on healthy HDF and A375 melanoma cells (Figure 10). For HDFs, despite an initial decrease in adaptation in the coated group on day 1 (**** *p* < 0.0001), cell viability recovered to 2D control levels by day 7. This demonstrates excellent long-term biocompatibility, a result of diatomite’s high porosity, which enhances protein adsorption and cell attachment [63]. Conversely, the scaffolds exerted a distinct suppressive effect on A375 cells. While the 2D control viability surged to ~180% by day 7, the uncoated PVA/diatomite group significantly restricted this aggressive proliferation (* *p* < 0.05). This biophysical phenomenon is attributed to the topographical barriers of the 3D nanofiber morphology and diatomite particles, which disrupt melanoma focal adhesions [64]. Most strikingly, the *Inula helenium*-coated group exhibited the maximum cytotoxic activity, keeping cancer cell viability at the statistically lowest level (* *p* < 0.05). This observed anti-tumoral effect is likely driven by sesquiterpene lactones (e.g., alantolactone) in Inula, which are known to induce apoptosis by inhibiting Wnt/β-catenin and STAT3 signaling pathways [65,66]. The synergy between topographical restriction and pharmacological inhibition suggests that these coated nanofibers have great potential as bioactive dressings for post-resection tumor margins, concurrently suppressing residual cancer cells and promoting healthy tissue regeneration.

To evaluate the dual impact of the scaffolds on healthy tissue regeneration and cancer cell motility, an in vitro scratch wound healing assay was performed (Figure 11). For healthy HDF cells, both uncoated and *Inula helenium*-coated PVA/diatomite nanofibers significantly accelerated migration compared to the 2D control at 24 h (* *p* < 0.05), with the coated group achieving almost complete wound closure (~100%). This enhanced migratory response can be attributed to the biomimetic nature of the scaffolds. Electrospun nanofibrous architectures closely mimic the native extracellular matrix (ECM), providing essential topographical cues that facilitate healthy fibroblast attachment and directed migration during the physiological wound healing process [67]. Interestingly, the migration behavior of A375 melanoma cells presented a complex response to the bioactive scaffolds. While the uncoated PVA/diatomite group slightly restricted A375 migration compared to the 2D control at 48 h, the *Inula helenium*-coated nanofibers significantly increased the wound closure rate of A375 cells at both 24 and 48 h (* *p* < 0.05 and *** *p* < 0.001, respectively). When evaluated alongside the MTT viability results, which demonstrated profound cytotoxicity against A375 cells, this enhanced migration on the Inula-coated scaffolds reveals a classic phenotype switching in melanoma cells [68]. When melanoma cells are exposed to localized pharmacological stress or complex 3D biomaterial microenvironments, they frequently alter their phenotypic state. In this state, they tend to halt proliferation to conserve energy while upregulating their motility to escape the hostile conditions [69]. This adaptive escape behavior aligns with broader cellular survival and resistance mechanisms frequently observed in other aggressive solid tumors under therapeutic pressure. For instance, Zehra K et al. (2024) recently demonstrated a comparable stress-adaptation phenomenon in colorectal cancer, where HT29 cells developed acquired resistance to capecitabine (CR/HT29) by significantly altering their proliferation and apoptotic pathways [36]. Notably, their study showed that introducing bioactive phytochemicals such as fisetin effectively modulated this resistant phenotype and induced apoptosis [36]. Similarly, in our study, the bioactive phytochemicals present in the *Inula helenium* coating exert profound pharmacological stress on A375 cells, forcing an acute stress-induced escape response rather than allowing the cells to develop long-term resistance. Ultimately, these findings suggest that *Inula helenium*-coated scaffolds hold great potential as localized bioactive dressings; they appear capable of supporting the rapid fibroblast migration necessary for healthy tissue repair, while actively stressing and suppressing residual melanoma cells in the surgical margin.

To further elucidate the molecular rationale behind these cellular responses, the enhanced motility of A375 melanoma cells on Inula-coated scaffolds must be distinguished from a pro-metastatic phenotype [68,69]. Under localized pharmacological stress—driven primarily by eudesmane-type sesquiterpene lactones (e.g., alantolactone and isoalantolactone)—aggressive melanoma cells undergo an acute stress-induced phenotype switching [65,68]. These bioactive compounds trigger intracellular reactive oxygen species (ROS) accumulation and block crucial oncogenic survival pathways, specifically inhibiting Wnt/β-catenin and STAT3 signaling cascades [24,66]. In response to this acute molecular blockade, melanoma cells temporarily arrest proliferation to conserve metabolic energy while transiently upregulating motility in an attempt to escape the hostile biomaterial microenvironment [68,69]. However, as confirmed by our quantitative MTT assay and Live/Dead fluorescent microscopy (Figure 10B and Figure 12), this migratory response is non-viable and short-lived. By day 3, the vast majority of A375 cells undergo extensive cell membrane compromise, nuclear fragmentation, and apoptotic death. Conversely, healthy human dermal fibroblasts (HDFs) possess robust baseline enzymatic antioxidant systems capable of neutralizing moderate phytochemical exposure. Furthermore, the 3D electrospun nanofibrous matrix paired with porous diatomite microstructures provides biomimetic extracellular matrix (ECM) topographical cues that promote healthy fibroblast adhesion, spreading, and migration without triggering apoptotic cascades [7,19]. Consequently, the hybrid platform effectively forces residual melanoma cells into an unsustainable escape response that terminates in apoptosis, while simultaneously supporting healthy fibroblast-mediated tissue repair.

To morphologically validate the quantitative findings obtained from the MTT and scratch assays, a Live/Dead fluorescent viability assay was performed (Figure 12). Microscopic evaluation revealed that healthy HDF cells exhibited strong, uniform green fluorescence across all three groups (2D control, uncoated PVA/diatomite, and Inula-coated PVA/diatomite). The complete absence of red (dead) signals and the preservation of a well-spread, confluent morphology in the coated group demonstrate that the *Inula helenium* extract does not impose toxic stress on healthy fibroblasts, thereby confirming the excellent biocompatibility of the scaffolds. In stark contrast, while A375 melanoma cells maintained their viability on the 2D control and uncoated scaffolds, a dramatic cytotoxic shift occurred in the *Inula helenium*-coated group. In this group, almost the entire cell population exhibited red fluorescence, indicative of extensive cell death, and the cells were observed to be morphologically rounded and fragmented. This contrasting cellular response clearly demonstrates the biomaterial’s selective cytotoxicity. On one hand, intense exposure to sesquiterpene lactones from the Inula extract disrupts the cytoskeletal architecture of melanoma cells, driving them toward cell death [65,66]. On the other hand, the same coating does not harm the vital functions of healthy HDF cells. Consequently, these visual findings corroborate that the material safely supports healthy fibroblast proliferation and tissue repair, while actively suppressing aggressive melanoma cells via its bioactive coating.

### 3.8. Antibacterial Activity Test

The antimicrobial activity of *Inula helenium* extracts was evaluated using the well diffusion method. The findings revealed that the extracts exhibited antibacterial activity against *S. aureus*. The well diffusion test results showed the formation of distinct inhibition zones against the tested bacterial strain. This indicates that *Inula helenium* extracts possess effective antibacterial potential against *S. aureus*. No antibacterial effect was observed against *P. aeruginosa* and *E. coli* bacteria (Figure 13 and Table 5). The antibacterial activity of PVA/diatomite nanofibers and *Inula helenium*-coated PVA/diatomite nanofibers was evaluated using a modified liquid microdilution method. The results of the experiment, as detailed in Table 6, demonstrated that *Inula helenium*-coated PVA/diatomite nanofibers strongly inhibited the growth of *S. aureus*. These results are consistent with those obtained when the extract was evaluated on its own. The lack of activity against Gram-negative bacteria may be due to the structural characteristics of the bacterial cell envelope. The main bioactive components of Inula helenium exhibit predominantly hydrophobic properties. In contrast, the outer membrane of Gram-negative bacteria is rich in lipopolysaccharide (LPS) and forms a highly hydrophilic barrier. This limits the passage of hydrophobic compounds through the outer membrane, reducing their concentration inside the cell and, consequently, preventing them from causing effective damage to the bacterial membrane. Furthermore, Gram-negative bacteria possess sophisticated multi-drug efflux pump systems. These pumps actively transport antimicrobial compounds out of the cell, reducing their intracellular accumulation and contributing to the bacteria’s reduced susceptibility to these compounds [70,71,72]. Literature on *Inula helenium* indicates that this plant exhibits significant antimicrobial activity, particularly against Gram-positive bacteria. In particular, it has been reported that sesquiterpene lactone derivatives exert a bactericidal effect by disrupting bacterial cell wall biosynthesis, and that this effect is particularly evident in *Staphylococcus aureus* strains. It is noted that plant extracts not only inhibit bacterial growth but also limit the progression of infection by suppressing virulence factors [73,74,75]. Furthermore, thanks to the phenolic and bioactive compounds it contains, *Inula helenium* accelerates epithelialization by increasing fibroblast activity and collagen synthesis, thereby exhibiting a regenerative effect that supports wound healing. The findings are consistent with the literature and suggest that *Inula helenium*-based bioactive wound dressings, unlike traditional dressing materials, may serve as a promising natural biomaterial capable of providing targeted antibacterial protection against Gram-positive pathogens like *S. aureus*, controlling inflammation, and supporting new tissue formation in wound microenvironments.

## 4. Conclusions

This study successfully developed a novel, bio-based “mineral botanical” hybrid nanofibrous dressing to address the complex requirements of post-surgical oncological wound management, thereby reducing the reliance on synthetic drugs. The core novelty and significance of this research lie in its successful transition from passive wound protection to an active, dual-function therapeutic interface through a highly effective “mineral-botanical” synergy. In conclusion, by synergizing the natural carrier capabilities and theoretical hemostatic potential of calcined diatomite with the therapeutic bioactivity of *Inula helenium*, this study provides strong preliminary in vitro evidence for a potential sustainable therapeutic platform. By offering a biobased alternative to synthetic agents, this strategy establishes a solid foundation for oncological wound management. As the present findings provide an encouraging in vitro proof of concept, future research will focus on evaluating the biological performance, local tissue integration, and wound healing efficacy of this hybrid platform using established in vitro, ex vivo, and/or in vivo models. This progressive translational pipeline will enable a thorough assessment of long-term bioactive release kinetics, local tumor recurrence suppression, and systemic biocompatibility, ultimately optimizing the dressing for clinical oncological wound management.

## Figures and Tables

**Figure 1:**
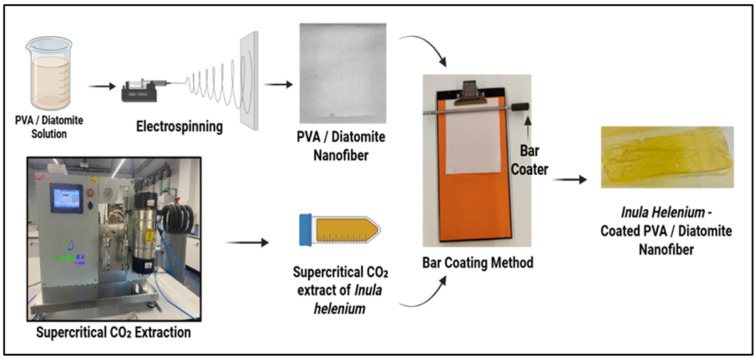
Fabrication of PVA/diatomite nanofibers and their coating with *Inula helenium* supercritical CO_2_ extract.

**Figure 2:**
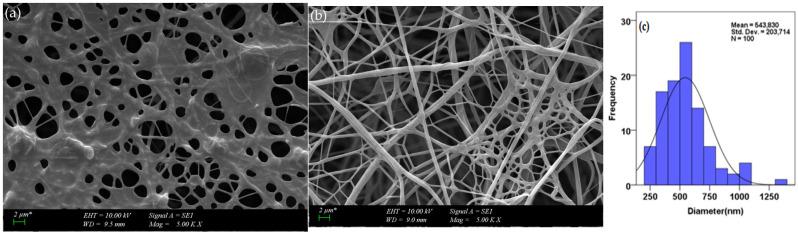
SEM images and diameter distribution of PVA/diatomite and *Inula helenium*–coated nanofibers: (**a**) *Inula helenium*–coated PVA/diatomite nanofibers at ×5000 magnification; (**b**) PVA/diatomite nanofibers at ×5000 magnification; (**c**) diameter distribution histogram of PVA/diatomite nanofibers.

**Figure 3:**
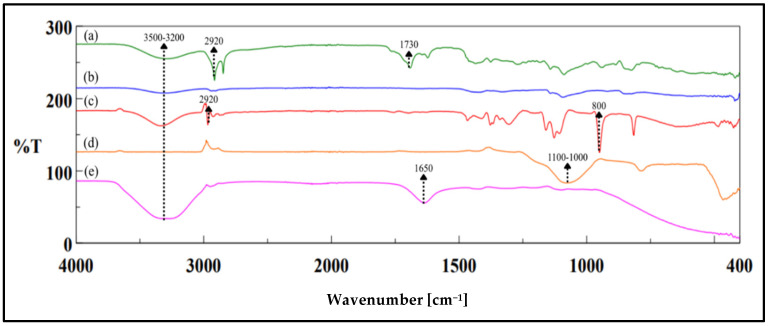
FTIR spectra of: (**a**) pure PVA, (**b**) calcined diatomite, (**c**) *Inula helenium* extract, (**d**) PVA/diatomite nanofiber mat, and (**e**) *Inula helenium*-coated PVA/diatomite nanofiber mat.

**Figure 4:**
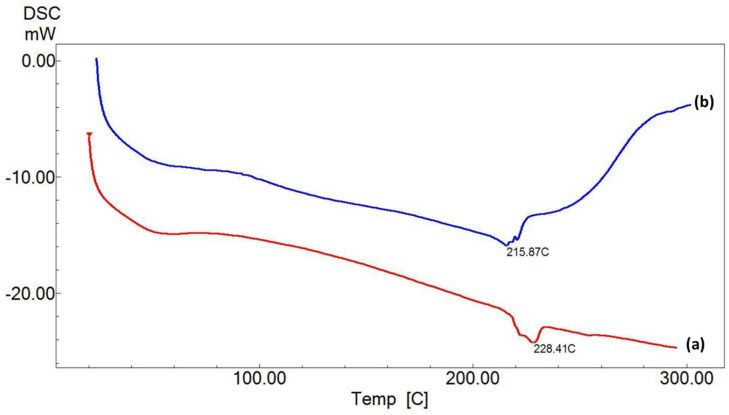
DSC thermogram of: (**a**) PVA/diatomite nanofiber mat and (**b**) *Inula helenium*-coated PVA/diatomite nanofiber mat.

**Figure 5:**
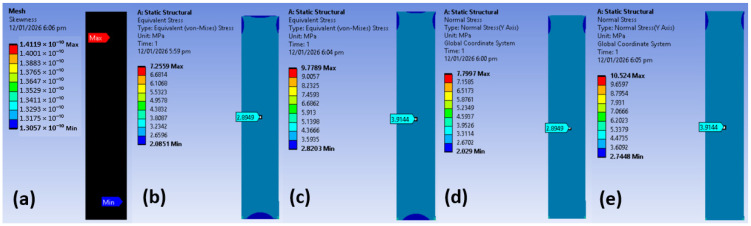
Numerical analysis contours: (**a**) mesh quality, (**b**) von Mises stress of the *Inula helenium*-coated PVA/diatomite nanofiber mat, (**c**) von Mises stress of the PVA/diatomite nanofiber mat, (**d**) normal stress of the *Inula helenium*-coated PVA/diatomite nanofiber mat, and (**e**) normal stress of the PVA/diatomite nanofiber mat.

**Figure 6:**
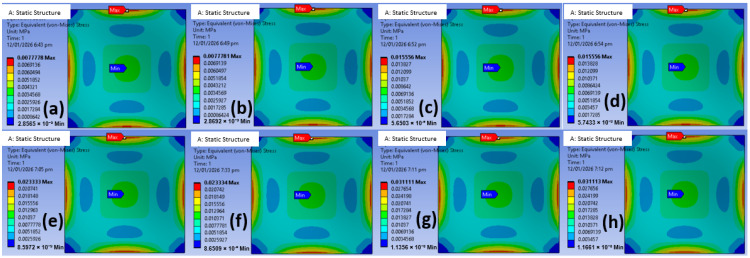
Equivalent stress distributions: (**a**) stress of the *Inula helenium*-coated PVA/diatomite nanofiber mat under 0.1 Pa, (**b**) stress of the PVA/diatomite nanofiber mat under 0.1 Pa, (**c**) stress of the *Inula helenium*-coated PVA/diatomite nanofiber mat under 0.2 Pa, (**d**) stress of the PVA/diatomite nanofiber mat under 0.2 Pa, (**e**) stress of the *Inula helenium*-coated PVA/diatomite nanofiber mat under 0.3 Pa, (**f**) stress of the PVA/diatomite nanofiber mat under 0.3 Pa, (**g**) stress of the *Inula helenium*-coated PVA/diatomite nanofiber mat under 0.4 Pa, and (**h**) stress of the PVA/diatomite nanofiber mat under 0.4 Pa.

**Figure 7:**
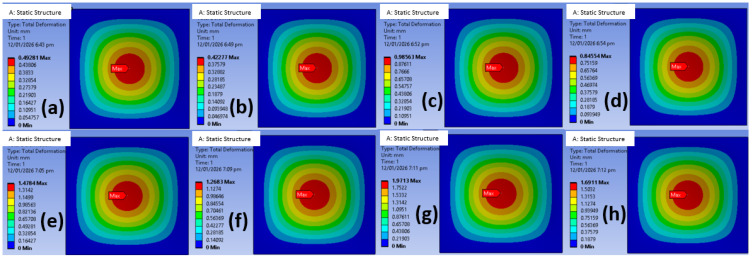
Total deformation distributions: (**a**) deformation of the *Inula helenium*-coated PVA/diatomite nanofiber mat under 0.1 Pa, (**b**) deformation of the PVA/diatomite nanofiber mat under 0.1 Pa, (**c**) deformation of the *Inula helenium*-coated PVA/diatomite nanofiber mat under 0.2 Pa, (**d**) deformation of the PVA/diatomite nanofiber mat under 0.2 Pa, (**e**) deformation of the *Inula helenium*-coated PVA/diatomite nanofiber mat under 0.3 Pa, (**f**) deformation of the PVA/diatomite nanofiber mat under 0.3 Pa, (**g**) deformation of the *Inula helenium*-coated PVA/diatomite nanofiber mat under 0.4 Pa, and (**h**) deformation of the PVA/diatomite nanofiber mat under 0.4 Pa.

**Figure 8:**
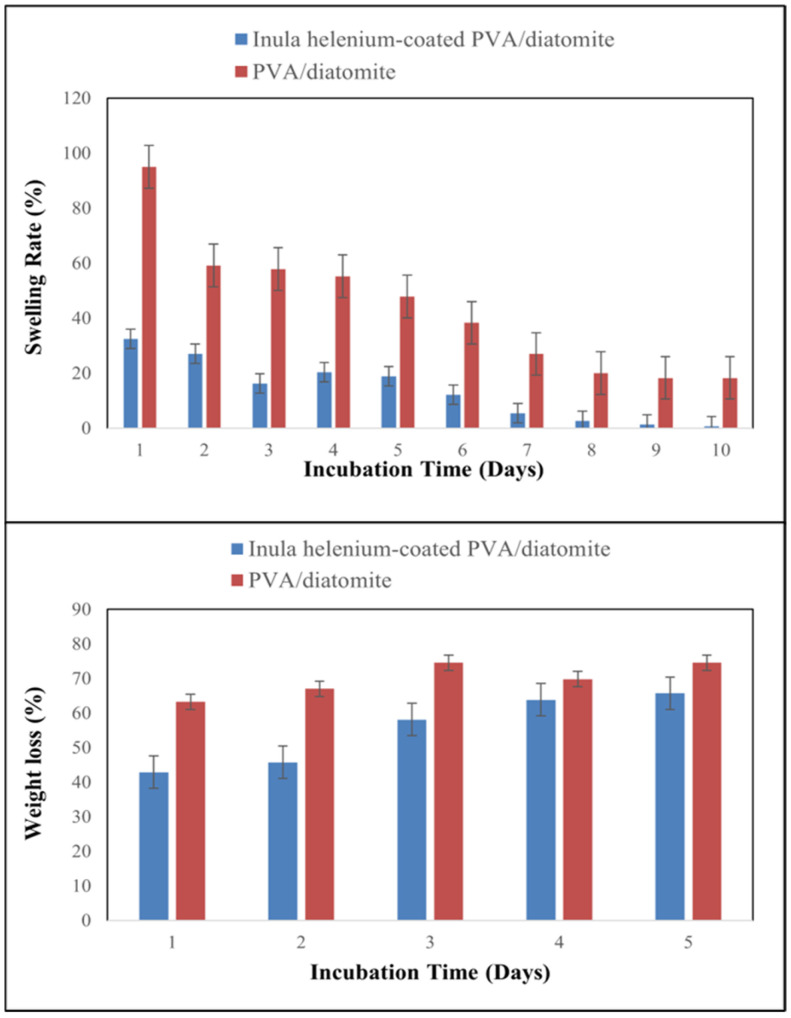
Swelling and degradation behavior of PVA/diatomite and *Inula helenium*-coated PVA/diatomite nanofibers.

**Figure 9:**
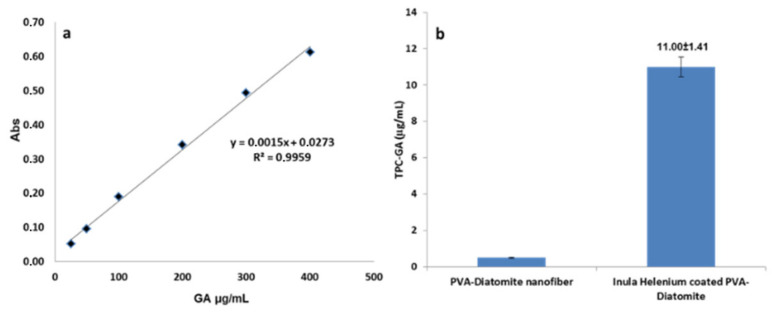
(**a**) Standard curve of Gallic acid; (**b**) total phenolic content of PVA/diatomite nanofiber.

**Figure 10:**
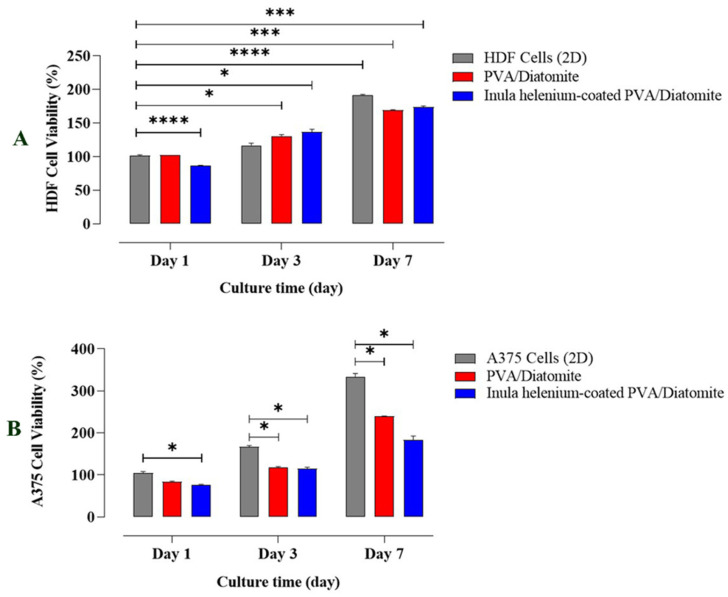
In vitro cell viability and proliferation of HDF and A375 cells in the presence of PVA/diatomite-based nanofibers compared to untreated 2D controls. (**A**) Quantitative analysis of HDF cell viability at days 1, 3, and 7. (**B**) Quantitative analysis of A375 cell viability at days 1, 3, and 7. Cell viability was evaluated using the MTT assay, and absorbance was measured at 570 nm. Data are presented as mean ± SEM (*n* = 3). Statistical significance was determined using ANOVA followed by Tukey’s multiple comparisons test (* *p* < 0.05, *** *p* < 0.001, **** *p* < 0.0001).

**Figure 11:**
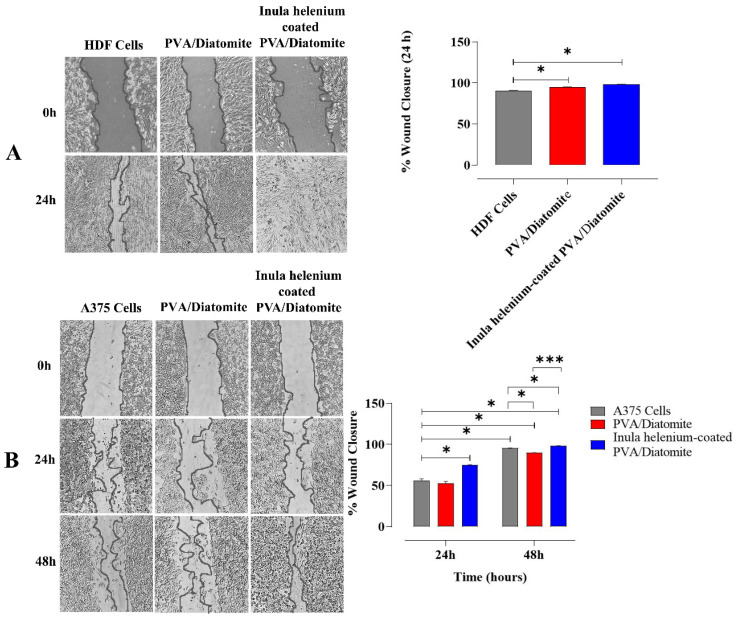
Wound healing capacity of HDF and A375 cells in the presence of PVA/diatomite-based nanofibers compared to the untreated 2D control. (**A**) Representative images of HDF cell monolayers at 0 h and 24 h after the scratch, and quantitative analysis of wound closure at 24 h. (**B**) Representative images of A375 cell monolayers at 0 h, 24 h, and 48 h after the scratch, and quantitative analysis of wound closure at 24 h and 48 h. Images were analyzed using ImageJ software, and the percentage of wound closure was calculated according to the standard formula. Data are presented as mean ± SEM (*n* = 3). Statistical significance was determined using one-way ANOVA followed by Tukey’s multiple comparisons test (* *p* < 0.05, *** *p* < 0.001).

**Figure 12:**
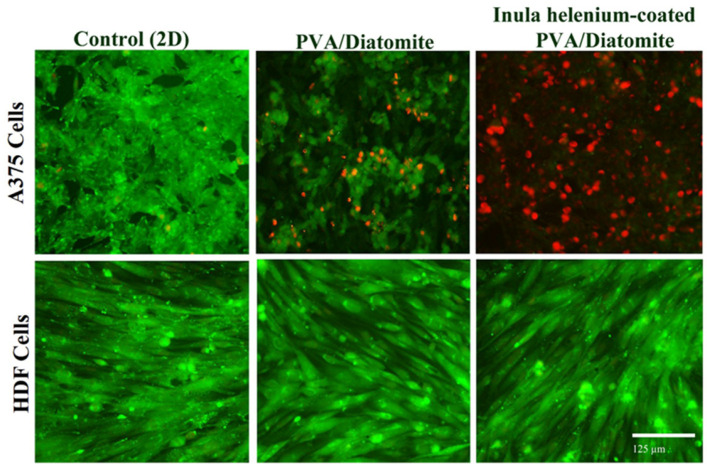
Fluorescence microscopy images showing the viability and morphology of HDF and A375 cells cultured on 2D controls, uncoated PVA/diatomite, and *Inula helenium*-coated PVA/diatomite nanofibers at day 3. Cells were evaluated using a Live/Dead assay, where viable cells exhibit green fluorescence and dead cells exhibit red fluorescence. The images demonstrate the excellent biocompatibility of the scaffolds with healthy HDF cells, as well as the distinct and selective cytotoxicity of the Inula-coated scaffolds against A375 melanoma cells. Scale bar: 125 µm.

**Figure 13:**
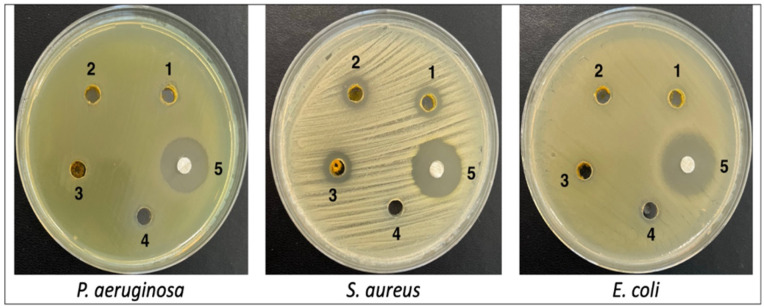
Antibacterial activities of *Inula helenium* extracts. 1. 17 mg/mL, 2. 34 mg/mL, 3. 68 mg/mL, 4. DMSO Control (It has been used as a solvent), and 5. Gentamicin (10 µg/disc).

**Table 1:** Mechanical properties of the nanofiber samples.

Samples	Max Force (N)	Max Stress (MPa)	Max Stroke (mm)	Max Strain (%)	Elastic Modulus
*Inula helenium*-coated PVA/diatomite	0.79 ± 0.03	2.94 ± 0.57	2.04 ± 0.64	4.05 ± 1.26	64.62 MPa
PVA/diatomite	1.10 ± 0.16	3.90 ± 0.31	6.75 ± 3.56	13.56 ± 7.33	75.33 MPa

**Table 2:** Experimental and numerical stresses.

Mats	Experimental Data, MPa	Numerical Data, MPa		% Diff
	Tensile Stress	Von Mises Stress	Normal Stress	
*Inula helenium*-coated PVA/diatomite	2.9384	2.8949	2.8949	±1.48
PVA/diatomite	3.9039	3.9144	3.9144	±0.27

**Table 3:** Equivalent stress and total deformation.

No	Pressure, Pa	Mat Type	Equivalent Stress, kPa	Total Deformation, mm
1	0.1	*Inula helenium*-coated PVA/diatomite	7.778	0.493
2	0.1	PVA/diatomite	7.778	0.423
3	0.2	*Inula helenium*-coated PVA/diatomite	15.556	0.986
4	0.2	PVA/diatomite	15.556	0.846
5	0.3	*Inula helenium*-coated PVA/diatomite	23.333	1.478
6	0.3	PVA/diatomite	23.333	1.268
7	0.4	*Inula helenium*-coated PVA/diatomite	31.111	1.971
8	0.4	PVA/diatomite	31.111	1.691

**Table 4:** ANOVA for equivalent stress and deformation.

Source	DF	Seq SS	Adj MS	% Effect	Seq SS	Adj MS	F	P	% Effect
Pressure	3	604.91	201.64	100	2.09398	0.69799	170.94	0.00	96.61
Mat Type	1	0	0	0	0.06125	0.06125	15.00	0.03	2.83
Error	3	0	0	0	0.01225	0.00408			0.57
Total	7	604.91		100	2.16748				100
The F-test is zero. R-Sq and R-Sq(adj) = 100.00%					R-Sq = 99.43% and R-Sq(adj) = 98.68%				

**Table 5:** Antibacterial activities of *Inula helenium* extracts: inhibition zone diameter (mm).

	17 mg/mL	34 mg/mL	68 mg/mL	DMSO Control	Gentamicin (10 µg/Disc)
*P. aeruginosa*	-	-	-	-	18 ± 0.5
*S. aureus*	10 ± 1	10 ± 1	11 ± 1	-	19 ± 0.5
*E. coli*	-	-	-	-	20 ± 0.5

**Table 6:** The antibacterial activities of the PVA/diatomite nanofiber and *Inula helenium*-coated PVA/diatomite nanofiber.

	AVG OD625	% Inhibition
*Staphylococcus aureus*-Control	1.558 ± 0.01	-
*Inula helenium*-coated PVA/diatomite nanofiber	0.09 ± 0.03	94%
PVA/diatomite nanofiber	1.662 ± 0.05	-
*Pseudomonas aeruginosa*-Control	1.349 ± 0.1	-
*Inula helenium*-coated PVA/diatomite nanofiber	1.177 ± 0.1	-
PVA/diatomite nanofiber	1.544 ± 0.08	-
*Escherichia coli*-Control	1.051 ± 0.03	-
*Inula helenium*-coated PVA/diatomite nanofiber	1.027 ± 0.005	-
PVA/diatomite nanofiber	1.070 ± 0.02	-

## Data Availability

The original contributions presented in the study are included in the article; further inquiries can be directed to the corresponding authors.

## References

[1] Mirhaj M., Labbaf S., Tavakoli M., Seifalian A.M. (2022). Emerging treatment strategies in wound care. Int. Wound J..

[2] Tang S. (2021). Dressings for Advanced Wound Care.

[3] Wild A., Shortall C., Dewachi O., Naim C., Green A., Hussain S., Abbara A. (2025). Conflict-associated wounds and burns infected with GLASS pathogens in the Eastern Mediterranean Region: A systematic review. BMC Infect. Dis..

[4] Olsson M., Järbrink K., Divakar U., Bajpai R., Upton Z., Schmidtchen A., Car J. (2019). The humanistic and economic burden of chronic wounds: A systematic review. Wound Repair Regen..

[5] Tuchin V.V., Genina E.A., Tuchina E.S., Svetlakova A.V., Svenskaya Y.I. (2022). Optical clearing of tissues: Issues of antimicrobial phototherapy and drug delivery. Adv. Drug Deliv. Rev..

[6] Alven S., Peter S., Mbese Z., Aderibigbe B.A. (2022). Polymer-Based Wound Dressing Materials Loaded with Bioactive Agents: Potential Materials for the Treatment of Diabetic Wounds. Polymers.

[7] Su Y., Toftdal M.S., Le Friec A., Dong M., Han X., Chen M. (2021). 3D Electrospun Synthetic Extracellular Matrix for Tissue Regeneration. Small Sci..

[8] Hao Q., Schossig J., Towolawi A., Xu K., Bayiha E., Mohanakanthan M., Savastano D., Jayaraman D., Zhang C., Lu P. (2024). High-Speed Electrospinning of Ethyl Cellulose Nanofibers via Taylor Cone Optimization. ACS Appl. Eng. Mater..

[9] Hao Z., Dong M., Wang Y., Wang Z., Zhang Z., Chen J., Chen R., Li Z., Wang J., Shi G. (2025). Advancing electrospun nanofiber scaffolds for next-generation tissue engineering: From trend analysis to multifunctionalization and hybrid fabrication. Mater. Horiz..

[10] Vigani B., Rossi S., Sandri G., Bonferoni M.C., Milanesi G., Bruni G., Ferrari F. (2018). Coated electrospun alginate-containing fibers as novel delivery systems for regenerative purposes. Int. J. Nanomed..

[11] Lima L.L., Taketa T.B., Beppu M.M., Sousa I.M.D.O., Foglio M.A., Moraes Â.M. (2019). Coated electrospun bioactive wound dressings: Mechanical properties and ability to control lesion microenvironment. Mater. Sci. Eng. C.

[12] Bikuna-Izagirre M., Aldazabal J., Paredes J. (2022). Gelatin Blends Enhance Performance of Electrospun Polymeric Scaffolds in Comparison to Coating Protocols. Polymers.

[13] Türkoğlu G.C., Khomarloo N., Mohsenzadeh E., Gospodinova D.N., Neznakomova M., Salaün F. (2024). PVA-Based Electrospun Materials—A Promising Route to Designing Nanofiber Mats with Desired Morphological Shape—A Review. Int. J. Mol. Sci..

[14] Zahra F.T., Quick Q., Mu R. (2023). Electrospun PVA Fibers for Drug Delivery: A Review. Polymers.

[15] Marcuello C., Foulon L., Chabbert B., Molinari M., Aguié-Béghin V. (2018). Langmuir–Blodgett Procedure to Precisely Control the Coverage of Functionalized AFM Cantilevers for SMFS Measurements: Application with Cellulose Nanocrystals. Langmuir.

[16] Zhao J., Wang P., Sun L., Sun S., Cao X. (2025). Cellulose nanocrystals reinforced polyvinyl alcohol hydrogel with thermal, mechanical, and ionic responsive behavior. Int. J. Biol. Macromol..

[17] Subramaniam T., Fauzi M.B., Lokanathan Y., Law J.X. (2021). The Role of Calcium in Wound Healing. Int. J. Mol. Sci..

[18] Tian G., Wang Z., Huang Z., Xie Z., Xia L., Zhang Y. (2024). Clays and Wound Healing. Materials.

[19] Rabiee N., Khatami M., Soufi G.J., Fatahi Y., Iravani S., Varma R.S. (2021). Diatoms with Invaluable Applications in Nanotechnology, Biotechnology, and Biomedicine: Recent Advances. Am. Chem. Soc..

[20] Przybek A., Łach M., Hebdowska-Krupa M., Miernik K., Mikuła J. (2024). Research of the physical and chemical properties of diatomite as a carrier of phase change materials for use in advanced building materials. Front. Mater..

[21] Mustafov S.D., Sen F., Seydibeyoglu M.O. (2020). Preparation and characterization of diatomite and hydroxyapatite reinforced porous polyurethane foam biocomposites. Sci. Rep..

[22] Yang G., Yang L., Xu F. (2024). Isoalantolactone: A review on its pharmacological effects. Front. Pharmacol..

[23] Petkova N., Vrancheva R., Mihaylova D., Ivanov I., Pavlov A., Denev P. (2015). Antioxidant activity and fructan content in root extracts from elecampane (*Inula helenium* L.). J. BioSci. Biotechnol..

[24] Cao F., Chu C., Qin J.-J., Guan X. (2023). Research progress on antitumor mechanisms and molecular targets of Inula sesquiterpene lactones. Chin. Med..

[25] Chun J., Park S.-M., Lee M., Ha I.J., Jeong M.-K. (2023). The Sesquiterpene Lactone-Rich Fraction of *Inula helenium* L. Enhances the Antitumor Effect of Anti-PD-1 Antibody in Colorectal Cancer: Integrative Phytochemical, Transcriptomic, and Experimental Analyses. Cancers.

[26] Adamu B.F., Gao J., Jhatial A.K., Kumelachew D.M. (2021). A review of medicinal plant-based bioactive electrospun nano fibrous wound dressings. Mater. Des..

[27] Fatehi P., Abbasi M. (2020). Medicinal plants used in wound dressings made of electrospun nanofibers. J. Tissue Eng. Regen. Med..

[28] Mouro C., Gouveia I.C. (2023). Electrospun wound dressings with antibacterial function: A critical review of plant extract and essential oil incorporation. Crit. Rev. Biotechnol..

[29] Uddin M.N., Ali A., Jobaer M., Mahedi S.I., Krishnamoorthy A., Bhuiyan M.A.R. (2024). Electrospun nanofibers based on plant extract bioactive materials as functional additives: Possible sources and prospective applications. Mater. Adv..

[30] Langwald S.V., Ehrmann A., Sabantina L. (2023). Measuring Physical Properties of Electrospun Nanofiber Mats for Different Biomedical Applications. Membranes.

[31] Pytel A., Kiusalaas J. (2012). Mechanics of Materials. https://repositori.mypolycc.edu.my/handle/123456789/5333.

[32] Cetin F.S., Avci T., Uygur E., Ilhan E., Kaya E., Tinaz G.B., Duta L., Dogan C., Gunduz O. (2025). Development and Characterization of Antimicrobial Chitosan/Polyethylene Oxide/Bacterial Cellulose Nanofibers. Polymers.

[33] Alkaya D.B., Seyhan S.A., Ozturk B.N. (2019). Influence of extraction method on antioxidant properties of *Rheum ribes* root extract. Ovidius Univ. Ann. Chem..

[34] Guldorum Y., Ayran M., Bulut B., Ilgar S., Ulag S., Kanli Z., Aydin B., Gulhan R., Bedir T., Gunduz O. (2024). Ethosuximide-loaded bismuth ferrite nanoparticles as a potential drug delivery system for the treatment of epilepsy disease. PLoS ONE.

[35] Daaboul M., Akkaya A., Kanli Z., Gunduz O., Aydın B., Alarcin E., Ozmen M.M., Topuzogullari M. (2025). Nanoparticle-Embedded GelMA/NIPAm Hydrogels: A Temperature-Responsive Hybrid System for Controlled Drug Release. Macromol. Biosci..

[36] Zehra K., Banu A., Can E., Hülya C. (2024). Fisetin and/or capecitabine causes changes in apoptosis pathways in capecitabine-resistant colorectal cancer cell lines. Naunyn-Schmiedeberg’s Arch. Pharmacol..

[37] Weinstein M.P. (2020). Performance Standards for Antimicrobial Susceptibility Testing.

[38] Akduman Ç. (2019). Fabrication and Characterization of Diatomite Functionalized Cellulose Acetate Nanofibers. AATCC J. Res..

[39] Bozdag M., Urek F., Cesur S., Sahin A., Gunduz O. (2025). Bovine serum albumin (BSA)-Loaded polyvinyl alcohol (PVA)/chitosan (CH) / hydroxyapatite (HA) electrospun nanofibers for bone tissue regeneration. J. Drug Deliv. Sci. Technol..

[40] Sulutas R.B., Cesur S., Seyhan S.A., Alkaya D.B., Sahin A., Ekren N., Gunduz O. (2024). Electrospun amygdalin and Inula helenium extract-loaded polylactic acid (PLA)/Polyvinylpyrrolidone (PVP) nanofibrous patches for colon cancer treatment: Fabrication, characterization and antitumour effect results. J. Drug Deliv. Sci. Technol..

[41] Irvin C.W., Satam C.C., Meredith J.C., Shofner M.L. (2019). Mechanical reinforcement and thermal properties of PVA tricomponent nanocomposites with chitin nanofibers and cellulose nanocrystals. Compos. Part A Appl. Sci. Manuf..

[42] Sultana S., Imran S., Naveed A., Hussain S., Khattak R., Shah L.A., Rehan K., Rehan I., Rehman M.U., Hashmat U. (2024). Fabrication of nano filler doped PVA/starch biodegradable composites with enhanced thermal conduction, water barrier and antimicrobial performance for food industry. Heliyon.

[43] Suleiman G.S.A., Zeng X., Chakma R., Wakai I.Y., Feng Y. (2024). Recent advances and challenges in thermal stability of PVA-based film: A review. Polym. Adv. Technol..

[44] Zemljič L.F., Glaser T.K., Plohl O., Anžel I., Šimat V., Čagalj M., Mežnar E., Malin V., Sterniša M., Možina S.S. (2022). Biomass-Derived Plant Extracts in Macromolecular Chitosan Matrices as a Green Coating for PLA Films. J. Funct. Biomater..

[45] Drago E., Campardelli R., Lagazzo A., Firpo G., Perego P. (2023). Improvement of Natural Polymeric Films Properties by Blend Formulation for Sustainable Active Food Packaging. Polymers.

[46] Salleh N.A.M., Afifi A.M., Zuki F.M., SalehHudin H.S. (2025). Enhancing mechanical properties of chitosan/PVA electrospun nanofibers: A comprehensive review. Beilstein J. Nanotechnol..

[47] Nykiel M., Korniejenko K., Setlak K., Melnychuk M., Polivoda N., Kozub B., Hebdowska-Krupa M., Łach M. (2024). The Influence of Diatomite Addition on the Properties of Geopolymers Based on Fly Ash and Metakaolin. Materials.

[48] Dobrosielska M., Dobrucka R., Brząkalski D., Kozera P., Martyła A., Gabriel E., Kurzydłowski K.J., Przekop R.E. (2023). Polyamide 11 Composites Reinforced with Diatomite Biofiller—Mechanical, Rheological and Crystallization Properties. Polymers.

[49] Chandler A., Schofield R.M., Mouthuy P.-A., Assender H.E. (2025). Stretch-Induced Microstructural Evolution of Electrospun Polycaprolactone Microfibers for Biomedical Applications. ACS Appl. Polym. Mater..

[50] Gedik B., Erdem M.A. (2025). Electrospun PCL membranes for localized drug delivery and bone regeneration. BMC Biotechnol..

[51] Metwally S., Ura D.P., Krysiak Z.J., Kaniuk Ł., Szewczyk P.K., Stachewicz U. (2021). Electrospun PCL Patches with controlled fiber morphology and mechanical performance for skin moisturization via long-term release of hemp oil for atopic dermatitis. Membranes.

[52] Pensa N.W., Curry A.S., Bonvallet P.P., Bellis N.F., Rettig K.M., Reddy M.S., Eberhardt A.W., Bellis S.L. (2019). 3D printed mesh reinforcements enhance the mechanical properties of electrospun scaffolds. Biomater. Res..

[53] Timoshenko S. (2012). Theory of Elastic Stability.

[54] Wang C.M., Wang C.Y., Reddy J.N. (2004). Exact Solutions for Buckling of Structural Members.

[55] Rungruangkitkrai N., Phromphen P., Chartvivatpornchai N., Srisa A., Laorenza Y., Wongphan P., Harnkarnsujarit N. (2024). Water Repellent Coating in Textile, Paper and Bioplastic Polymers: A Comprehensive Review. Polymers.

[56] Tamburaci S., Kimna C., Tihminlioglu F. (2019). Bioactive diatomite and POSS silica cage reinforced chitosan/Na-carboxymethyl cellulose polyelectrolyte scaffolds for hard tissue regeneration. Mater. Sci. Eng. C.

[57] Peñas M.I., Criado-Gonzalez M., de Ilarduya A.M., Flores A., Raquez J.-M., Mincheva R., Müller A.J., Hernández R. (2023). Tunable enzymatic biodegradation of poly(butylene succinate): Biobased coatings and self-degradable films. Polym. Degrad. Stab..

[58] Rastogi V.K., Samyn P. (2015). Bio-Based Coatings for Paper Applications. Coatings.

[59] Wang J., Zhao Y.-M., Tian Y.-T., Yan C.-L., Guo C.-Y. (2013). Ultrasound-Assisted Extraction of Total Phenolic Compounds from *Inula helenium*. Sci. World J..

[60] Alan Y., Yılmaz N., Cabuk M. (2024). Investigation of phenolic contents and in vitro biological activities of *Inula helenium* subsp. pseudohelenium extracts. Acta Aliment..

[61] Sevindik E., Aydin S., Paksoy M.Y., Sokmen B.B. (2020). Anti-urease, total phenolic content and antioxidant activities of some Inula l. (Asteraceae) taxa in Turkey. Genetika.

[62] Esin E., Türker S.T. (2025). Optimization of Solvent Concentration in Phenolic Compound Extraction from *Inula helenium* L. Leaves Using D-Optimal Mixture Design. Hittite J. Sci. Eng..

[63] Terracciano M., Shahbazi M.-A., Correia A., Rea I., Lamberti A., De Stefano L., Santos H.A. (2015). Surface bioengineering of diatomite based nanovectors for efficient intracellular uptake and drug delivery. Nanoscale.

[64] Kumar S.U., Matai I., Dubey P., Bhushan B., Sachdev A., Gopinath P. (2014). Differentially cross-linkable core–shell nanofibers for tunable delivery of anticancer drugs: Synthesis, characterization and their anticancer efficacy. RSC Adv..

[65] Seca A.M., Grigore A., Pinto D.C., Silva A.M. (2014). The genus Inula and their metabolites: From ethnopharmacological to medicinal uses. J. Ethnopharmacol..

[66] Chen J., Zhang L., Chen J., Chen Y., Zou D., Pu Y., Wei M., Huang Y., Li Y., Huang Q. (2023). Alantolactone Inhibits Melanoma Cell Culture Viability and Migration and Promotes Apoptosis by Inhibiting Wnt/β-Catenin Signaling. Anti-Cancer Agents Med. Chem..

[67] Xue J., Wu T., Dai Y., Xia Y. (2019). Electrospinning and Electrospun Nanofibers: Methods, Materials, and Applications. Am. Chem. Soc..

[68] Arozarena I., Wellbrock C. (2019). Phenotype plasticity as enabler of melanoma progression and therapy resistance. Nat. Rev. Cancer.

[69] Nomdedeu-Sancho G., Gorkun A., Mahajan N., Willson K., Schaaf C.R., Votanopoulos K.I., Atala A., Soker S. (2023). In Vitro Three-Dimensional (3D) Models for Melanoma Immunotherapy. Cancers.

[70] Maher C., Hassan K.A. (2023). The Gram-negative permeability barrier: Tipping the balance of the in and the out. mBio.

[71] Thakur V., Uniyal A., Tiwari V. (2021). A comprehensive review on pharmacology of efflux pumps and their inhibitors in antibiotic resistance. Eur. J. Pharmacol..

[72] Nazzaro F., Fratianni F., De Martino L., Coppola R., De Feo V. (2013). Effect of Essential Oils on Pathogenic Bacteria. Pharmaceuticals.

[73] Buza V., Niculae M., Hanganu D., Pall E., Burtescu R.F., Olah N.-K., Matei-Lațiu M.-C., Vlasiuc I., Iozon I., Szakacs A.R. (2022). Biological Activities and Chemical Profile of *Gentiana asclepiadea* and *Inula helenium* Ethanolic Extracts. Molecules.

[74] O’shea S., Lucey B., Cotter L. (2009). In vitro activity of *Inula helenium* against clinical *Staphylococcus* aureus strains including MRSA. Br. J. Biomed. Sci..

[75] Qiu J., Luo M., Wang J., Dong J., Li H., Leng B., Zhang Q., Dai X., Zhang Y., Niu X. (2011). Isoalantolactone protects against Staphylococcus aureus pneumonia. FEMS Microbiol. Lett..

